# Intravascular Lymphoma-Associated Stroke: A Systematic Review of Case Studies

**DOI:** 10.7759/cureus.50896

**Published:** 2023-12-21

**Authors:** Riwaj Bhagat, Asna Shahab, Yukesh Karki, Samip Budhathoki, Mikki Sapkota

**Affiliations:** 1 Neurology, Conemaugh Memorial Medical Center, Johnstown, USA; 2 Internal Medicine, Conemaugh Memorial Medical Center, Johnstown, USA; 3 Internal Medicine, Kathmandu Medical College, Kathmandu, NPL; 4 Neurology, Geisinger Medical Center, Danville, USA

**Keywords:** cerebro-vascular accident (stroke), stroke, ivl, large b-cell diffuse lymphoma, intravascular lymphoma

## Abstract

Intravascular lymphoma (IVL) is an aggressive systemic large B-cell lymphoma that is a rare cause of stroke. The clinical characteristics of stroke associated with IVL remain underexplored, contributing to diagnostic complexities and a high mortality rate. This study endeavors to elucidate the salient clinical and investigative features of stroke linked to this condition. A systematic review was performed using the PubMed database from the incident to August 2023 including search categories for IVL and stroke. All studies, excluding review articles, were included in this study. There were 58 cases with a confirmed diagnosis of IVL associated with stroke, with a mean age of 62.9 ± 9.6 years (female 50%). Classical lateralizing stroke symptoms were noted in only 69% of cases. Other clinical syndromes included altered sensorium (31%), rapidly progressive cognitive impairment (23%), seizures (22%), and gait disturbances (19%). Common hematological abnormalities included elevated lactate dehydrogenase (LDH, 97%), erythrocyte sedimentation rate (ESR, 79%), C-reactive protein (CRP, 61%), interleukin-2, microglobulins, and cerebrospinal fluid (CSF) protein. CSF flow cytometry was not diagnostic, and cytology was mostly negative. The dynamic pattern for DWI/T2 lesions was predominant and primarily located in the subcortical regions. Diffuse background slowing (64%) was a major finding in the electroencephalogram. Seventy-one percent of cases died (n=45) mostly due to delayed diagnosis. Only 31% were treated with first-line R-CHOP (rituximab, cyclophosphamide, doxorubicin hydrochloride, vincristine, prednisone) chemotherapy, among whom 25% died. This study suggests that IVL-associated strokes carry a high mortality rate, largely due to challenges in timely diagnosis and therapy. Unlike classical stroke syndrome, key indicators to aid in early diagnosis include a clinical syndrome of multiple non-lateralizing neurological symptoms, dynamic MRI DWI/T2-lesions primarily located in subcortical regions, elevated serum LDH, ESR, CRP, interleukins, microglobulin, CSF protein, and CSF polymerase chain reaction analysis, apart from tissue examination. Larger studies should be performed to establish diagnostic and predictive scores.

## Introduction and background

Intravascular lymphoma (IVL) is a rare, aggressive systemic diffuse large B-cell lymphoma (DLBCL) in which lymphoma cells selectively involve lumina of vessels, particularly small and medium-sized, and rarely involve parenchyma [1,2]. It was first described as “angiotropic lymphoma” by Pfleger and Tappeiner in 1959 [3]. IVL is distinct from primary central nervous system lymphoma as it lacks the expression of adhesion molecules and matrix metalloproteinases, which are needed for the invasion of tumor cells from blood vessels into the brain parenchyma. Thereby, IVL is usually contained in the blood vessel lumen [4]. The lumen of blood vessels not only serves as a vehicle for dissemination but also as an active site for replication [5]. Based on clinical features, IVL is classified as a “classical” or Western variant, which mainly involves the central nervous system and skin, and an “Asian” variant, which predominantly presents as hemophagocytic syndrome [6]. Tumor cells can aggressively proliferate within the lumen, leading to its occlusion and ischemic stroke. Nevertheless, IVL is associated with a myriad of clinical syndromes, with cognitive impairment being a notable manifestation, while stroke symptoms make up only 8% of its symptomatic profile [7]. Detection of specific cell surface glycoproteins aids in the diagnosis; however, tissue biopsy with demonstration of lymphoid cells within the blood vessel lumen is essential for a definitive diagnosis [1,5]. The first line treatment for IVL is R-CHOP (rituximab, cyclophosphamide, doxorubicin hydrochloride, vincristine, prednisone) combination chemotherapy with a remission rate of 43-53% [8,9]. Due to the atypical presentations of IVL-associated strokes leading to diagnostic challenges, we aim to investigate the demographics, clinical features, investigative findings, and management of strokes associated with IVL. We hypothesized that strokes associated with IVL have specific clinical, laboratory, and radiological markers that can aid in early diagnosis.

## Review

Methods

We performed a systematic review according to the Preferred Reporting Items for Systematic Reviews and Meta-Analyses (PRISMA) guidelines. The PubMed database was searched using the Medical Subject Headings (MeSH) terms and keywords from the incident to August 2023 to find the relevant studies in English literature. The search strategy used is shown in Table 1. Relevant references included in the articles searched were added for review.

**Table 1:** Search strategy for electronic databases

Search Strategy	Database
(("Intravascular"[All Fields] AND ("large"[All Fields] OR "largely"[All Fields] OR "larges"[All Fields]) AND ("lymphoma, b cell"[MeSH Terms] OR ("lymphoma"[All Fields] AND "b cell"[All Fields]) OR "b-cell lymphoma"[All Fields] OR "b cell lymphoma"[All Fields])) OR ("Intravascular"[All Fields] AND ("lymphoma"[MeSH Terms] OR "lymphoma"[All Fields] OR "lymphomas"[All Fields] OR "lymphoma s"[All Fields]))) AND ("stroke"[MeSH Terms] OR "stroke"[All Fields] OR "strokes"[All Fields] OR "stroke s"[All Fields] OR ("hemorrhagic stroke"[MeSH Terms] OR ("hemorrhagic"[All Fields] AND "stroke"[All Fields]) OR "hemorrhagic stroke"[All Fields]) OR ("ischemic stroke"[MeSH Terms] OR ("ischemic"[All Fields] AND "stroke"[All Fields]) OR "ischemic stroke"[All Fields]))	PubMed

Inclusion and Exclusion Criteria

All human studies associated with IVL and stroke, including case reports, case series, observational studies, clinical trials, editorials, and opinion articles, were included. All non-human studies, IVL not associated with stroke, and review articles were excluded.

Outcome of Interest

Data regarding demographics, clinical features, neuro-imaging features, laboratory findings, management, and prognosis were extracted. Primary outcomes were the frequency of the above-outlined variables.

Statistical Analysis

Descriptive analysis was performed using the SAS software (StataCorp 18.0, USA). Kempen et al. reporting guidelines were followed for reporting the systematic review of case reports [10].

Results

Study Selection 

**Figure 1:**
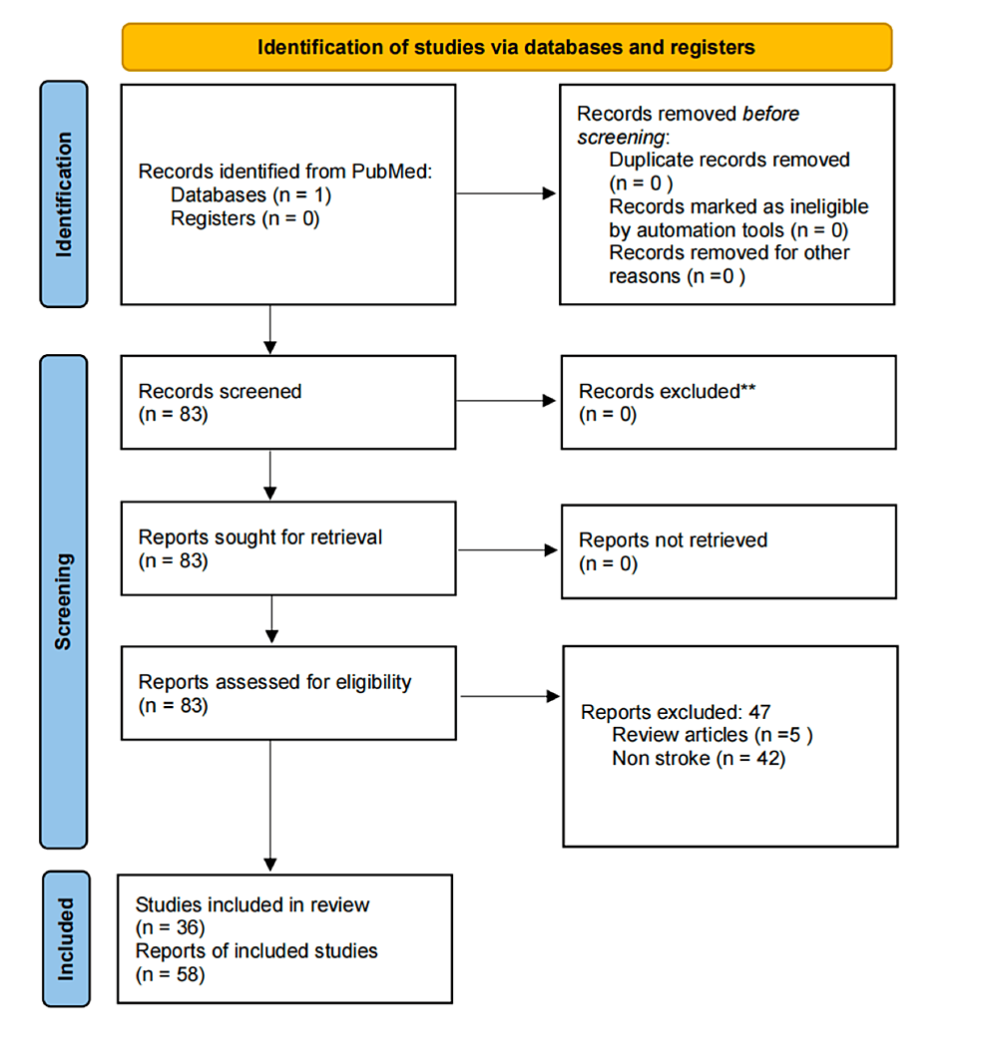
PRISMA flowchart Preferred Reporting Items for Systematic Reviews and Meta-Analyses (PRISMA) 2020 flowchart showing study selection

A total of 83 studies were identified through a database search, as shown in Figure 1. Forty-seven articles were excluded, among which five were review articles and 42 were not associated with stroke. A total of 36 studies met the inclusion criteria and were included in the review. All of the 36 studies were either case reports or series.

Study Characteristics

From 36 studies, there was a cohort of 58 cases with a confirmed diagnosis of IVL associated with stroke. The mean age was 62.9 ± 9.6 years, of which 50% were female. The diagnosis of the IVL was confirmed mostly from the tissue biopsy, except in two cases, which were confirmed from cerebrospinal fluid (CSF) polymerase chain reaction (PCR) analysis [11]. The characteristics of the cases are presented in Table 2.

**Table 2:** Clinical, laboratory, imaging and biopsy findings, treatment, and prognosis of all case reports IVL: intravascular lymphoma, MRI: magnetic resonance imaging, MRA: magnetic resonance angiography, CT: computed tomography, EEG: electroencephalogram, DSA: digital subtraction angiography, CSF: cerebrospinal fluid, LDH: lactate dehydrogenase, ESR: erythrocyte sedimentation rate, CRP: C-reactive protein, sIL-2R: soluble interleukin-2 receptor, PCR: polymerase chain reaction, IPH: intraparenchymal hemorrhage, SAH: subarachnoid hemorrhage, FDG: fluorodeoxyglucose, PET: positron emission tomography, CAP: chest, abdomen, and pelvis, CTAP: computed tomography arterial portography

Author/year	Age (years)/sex	Clinical features	Serum and CSF findings	Imaging, EEG, and biopsy findings	Treatment and prognosis
Glass et al. (1993) [12]	62 M	Initially presented with progressive lethargy and cognitive impairment. Later developed left-sided weakness	Serum: elevated LDH, thrombocytopenia	MRI brain: Multiple T2 hyperintense white matter lesions; quadriceps muscle biopsy: lymphoma cells in small blood vessels	Initially treated with plasmapheresis and later with cyclophosphamide. Died after 12 months
	63 M	Initially presented with dysarthria, gait problem, cognitive impairment, headache, dizziness, dystonia. Later developed acute onset left-sided weakness and flaccid paraplegia	Serum: thrombocytopenia; CSF: elevated protein and 5 lymphocytes/mm^3^	CT head: hypodense lesion in septum pellucidum and corpus callosum; CT head after 2 months: periventricular hypodensities and ventriculomegaly; brain biopsy: bland infarction with lymphoma cells in blood vessels lumen, perivascular space, and extravascular foci in corpus callosum	Treated with dexamethasone and whole brain radiation. Died after 2 months
	58 F	Initially presented with progressive gait disturbance, dysarthria, and left-sided paresthesia. 2 months later developed paraplegia and urine incontinence	CSF: elevated protein, 6 leukocytes/mm^3^	CT head: hypodense lesion in right motor strip; CT head after 2 months: multiple while matter hypodensities; conventional angiogram: left MCA distal occlusion; brain biopsy: ischemic damage, with lymphoma cells within small blood vessels	Treated with steroids, radiation, and chemotherapy. Died after 1 year
Bergmann et al. (1994) [13]	58 F	Initially presented with 1 month of fatigue and night sweats. 3 months later developed an acute onset of right hemiparesis, gait disturbances, limb paresthesia, and cognitive slowing. 4 months later developed transient aphasia, visual symptoms, and a fever of unknown origin	Serum: ESR 30/hr, CRP 48 mg/l, LDH 385 U/I, elevated alfa 1-2, g-globulin; 1 month later: ESR 80/hr, LDH 5843 U/I	MRI brain: multiple T2 hyperintense lesions of centrum semiovale in the occipital region, enhancing lesions in occipital lobe white matter, lacune in right periventricular region, ventriculomegaly; vessel imaging: normal; SPECT: normal; EEG: normal; bone marrow biopsy: myelofibrosis; splenic aspirate: negative; left quadriceps biopsy: IVL cells; brain biopsy: multiple infarcts with occlusion of small vessels by neoplastic cells mixed with fibrin	Treated with steroids. Died after 8 months
	54 M	Initially presented with seizure, confusion, left left-sided hemiparesis. 1 month later developed status epilepticus	Serum: ESR 29/hr, LDH 942 U/I, alfa-2-globulin 11%	CT head: right pons hypodense lesion; EEG: diffuse slowing brain biopsy: multiple hemorrhages and necrosis in right pons with occlusion of small vessels by neoplastic cells	Died after 2 months from respiratory failure
	56 M	Initially presented with gait disturbances, dizziness, left-sided paresis, sensory loss, and ataxia. Later developed a fever of unknown origin, left eye ptosis, and became comatose	Serum: elevated ESR and alfa-globulin; CSF: protein 91 g/dl, cells 31/microl	CT head: hypodense lesion in right parietal and bilateral occipital lobes; cerebral angiography: negative; EEG: diffuse slowing; brain biopsy: multiple recent and older infarcts and small vessel occlusion by neoplastic cells	Died after 2 months
	72 M	Initially presented with transient visual symptoms, right arm weakness. 1 month later developed right-sided weakness, aphasia, and dysarthria. Later developed seizures and renal and hepatic failure	Serum: elevated ESR, anemia, LDH 256 U/I	CT head: hypodense lesion in the right parieto-occipital region; MRI brain: multiple ischemic strokes in the subcortical region; EEG: diffuse slowing; brain biopsy: positive for infarcts with intravascular tumor cells	Died after 6 months
	71 M	Initially presented with 6 weeks of tremor, confusion, fever of unknown origin, gait disturbances, bilateral ataxia, dysarthria, and generalized myoclonus	Serum: elevated ESR, elevated CRP, anemia, LDH-800 U/I; CSF: protein 95 g/dl, cells 6/microl	CT head: unremarkable; EEG: diffuse slowing; bone marrow biopsy: lymphoid neoplasia; brain biopsy: microinfarcts with intravascular tumor cells	Died after 2 months
Heinrich et al. (2005) [14]	80 M	Rapidly progressive dementia, psychosis, and dysphasia over 1 week	Serum: ESR 25 mm/hr, elevated LDH; CSF: lymphocytic pleocytosis with 7 cells/microL, protein 1190 mg/dl, albumin (CSF/serum) 21, elevated neuron-specific enolase 62.5 microgram/L and S-100 4.95 microgm/L	MRI brain: DWI hyperintense along with T2 hyperintense small periventricular, cortical-subcortical lesions; vessel imaging: no significant stenosis; brain biopsy: lymphoma cells in the vascular wall of small and medium-sized leptomeningeal and intracerebral blood vessels	Died on the 10^th^ day of admission
Baehring et al. (2005) [15]	39 F	Progressive cognitive decline, headache, ataxia, and left homonymous hemianopia	NA	MRI brain: acute infarction along with enhancing cortical and sub-cortical lesions; brain biopsy: IVL	NA
	60 M	Lethargy	NA	MRI brain: multiple cortical DWI hyperintense lesion; gastrocnemius muscle biopsy-IVL	NA
	54 F	Progressive diminished vision with floaters	NA	MRI brain: resolving DWI and T2 hyperintense lesions in bilateral subcortical regions and few contrast-enhancing lesions, left optic nerve T2 hyperintensity; skin biopsy-IVL	NA
	79 M	Progressive quadriparesis, lower limb numbness over 1 month along with neurogenic bladder	CSF: clonal IgH gene rearrangement	MRI brain: multifocal subcortical small vessel infarcts	NA
Immanura et al. (2006) [16]	75 F	Acute onset dysarthria and dysphagia. Symptoms resolved after ozagrel (anti-platelet). A month later, had acute aphasia that resolved with argatroban therapy. Later developed AMS with mild fever	Serum: elevated LDH, beta-2-microglobulin, interleukin-2; CSF: normal	Initial MRI brain: T2 lesion in the white matter of left frontal lobe; repeat MRI brain 1 month later: Diffuse T2 lesions in the white matter of bilateral frontal, temporal, and parieto-occipital lobes, with gyri form enhancement in bilateral temporal lobes and left parieto-occipital lobe; bone marrow biopsy: negative	Anti-platelets and steroids. Died after 10 months
Holmoy et al. (2007) [17]	62 M	Vertigo, visual hallucination, diplopia, left-sided hearing loss, aphasia. Over three weeks, added cognitive impairment, apraxia, and color blindness	NA	MRI brain: T2 hyperintense lesion in bilateral cerebellum and patireto-occipital regions; brain biopsy: lymphoma filling the lumen of small vessels, multiple ischemic infarcts	Treated with high-dose steroids if possible. Died after 3 weeks
Iijima et al. (2007) [18]	64 M	Dysarthria, right-hand weakness, low-grade fever over 3 months	Serum: elevated LDH, elevated CRP, anemia, thrombocytopenia; CSF: normal	MRI brain: small multifocal T2 hyperintense lesion along with DWI changes in the bilateral subcortical and cerebellar region; MRI brain 2 months later: similar new lesions in the bilateral subcortical region; MRI brain 1 year later: GRE lesion in the bilateral posterior cortical region and contrast-enhancing lesion in left periventricular parietal lobe; cerebral angiogram: normal; brain biopsy: atypical lymphoid cells in lumen of small vessels	Treated with six courses of chemotherapy (THP-CVP) with marked improvement and residual moderate dementia. Later, treated with radiation therapy for new lesions with improvement in lesion size
Anda et al. (2008) [19]	69 F	Left-hand weakness and left-face numbness. Initially treated with anticoagulation for suspected cardio-embolism. Later developed transient left-sided weakness and anti-platelets were added. Later, developed flaccid left-sided weakness	NA	Initial MRI brain: right frontal infarction and small T2 hyperintense lesion in right occipital lobe; initial MRA showed no intracranial vessel abnormalities; later, CT head: right frontal IPH and associated SAH when the patient developed flaccid left-sided weakness; DSA showed distal M1 aneurysm; brain biopsy: lymphoma cells in initial layer of aneurysm wall, no cerebral neoplastic cells	Anti-platelets and anti-coagulants. Left distal M1aneurysm was resected and superficial temporal artery-MCA anastomosis was performed. The patient died after 71 days of initial infarction which was followed by IPH
Sips et al. (2009) [20]	77 M	Progressive cognitive decline, fatigue, multiple episodes of transient stroke-like symptoms (right-sided numbness, dysarthria), and one episode of seizure	Serum: pancytopenia, gammopathy; CSF: elevated protein, negative flow cytometry	MRI brain: T2 hyperintense lesions in right frontal lobe, left temporal lobe, left cerebellar hemisphere, and left periventricular region; Repeat MRI brain 6 months later: resolution of IVL lesion; conventional angiogram: multifocal stenosis; EEG: diffuse slowing; bone marrow biopsy: myelodysplastic features; brain biopsy: occlusion of blood vessels by lymphoid cells	Treated with CHOP-R therapy. 6 months after patient had no neurological deficits
Sumer et al. (2009) [21]	54 M	Two days after coronary artery bypass surgery, developed visual hallucinations, insomnia, behavior changes, and gait problems	Serum: elevated ESR, elevated LDH, leukocytosis, and renal failure; blood smear: negative; CSF: normal	MRI brain: multiple DWI and T2 flair lesions in the bilateral white matter; repeat MRI brain: new subcortical T2 and DWI hyperintense lesions with enlargement of old lesions; conventional angiogram: negative; brain biopsy: multiple petechial hemorrhage with lymphoma cells within blood vessels	Treated with high-dose steroids and one dose of cyclophosphamide. The patient died on the 45^th^ day from cardio-respiratory failure
Boslooper et al. (2010) [22]	67 M	Progressive dysarthria and balance disturbances	Serum: elevated ESR 44mm/hr, elevated CRP 34 mg/L, elevated LDH 728 IU/L	CT head: multiple small subcortical infarctions; MRI brain: widespread multiple infarcts looking white matter T2 hyperintense lesions; repeat MRI brain: new subcortical T2 hyperintense lesions; FDG-PET: increased adrenal uptake; adrenal biopsy: IVL; bone marrow biopsy: negative	Treated with R-CHOP and intrathecal methotrexate. Complete remission after 6 cycles of chemotherapy
Yamada et al. (2010) [23]	65 M	Dysarthria and right-sided hemiparesis	NA	MRI brain: acute left hemispheric diffusion-restriction; IMP-SPECT: increased left frontal uptake; brain biopsy: IVL	Treated with whole brain radiation and steroids. The patient died after 7 months
	46 M	Transient dysarthria and left hemiparesis. 26 hours later developed an altered mental status and their left hemiparesis worsened. 6 days later developed paraplegia and respiratory failure	NA	MRI brain: DWI hyperintensity in right insular cortex; repeat MRI brain: continued new infarction; vessel imaging: multiple stenosis of right MCA; FDG-PET on 10^th^ day: high uptake in right temporal lobe; bone marrow and scalp biopsy: negative	Treated with R-CHOP and whole brain radiation. Clinical symptoms dramatically improved and a further 7 cycles of R-CHOP therapy were given
Hundsberger et al. (2011) [24]	56 M	Progressive cognitive impairment, disorientation, left-sided hemiparesis, and symptomatic epilepsy over 8 months. Later developed acute onset of worsening of left-sided hemiparesis	Serum: elevated LDH 355 U/L; CSF: normal protein, borderline pleocytosis; repeat CSF: elevated protein 68 mg/dl	Initial MRI brain: disseminated subcortical T2 hyperintense lesions; repeat MRI brain: acute and subacute infarction in multiple vascular territories; repeat MRI brain: new multiple arterial territory stroke; conventional angiogram: negative; brain biopsy: IVL	Treated with steroids and tapering-induced encephalopathic syndrome. Later treated with a combination of antiplatelets, anticoagulation, and high-dose steroids. After diagnosis of IVL, treated with CHOP-R X 6 cycles leading to dramatic improvement of neurological symptoms and complete resolution after 26 months
	61 F	Acute onset left hemiparesis, right hemianopia, and seizure. Weight loss of 10 kg over 6 months. After 4 weeks developed conus medullaris syndrome	Serum: thrombocytopenia 131 gm/L, elevated LDH 580 U/L and elevated liver enzymes; CSF: negative	CT head perfusion: perfusion defect in left hemisphere; MRI brain: large wedge-shaped T2 hyperintense lesion in right parietal lobe and pons; abdominal skin biopsy: negative; spleen biopsy: IVL	Initial with suspicion of acute stroke and symptomatic seizure, treated with thrombolytics and anti-epileptics. With suspicion of autoimmune encephalitis, treated with steroids, with improvement of symptoms. After conus medullaris syndrome, treated with plasmapheresis. Treated with CHOP-R therapy after diagnosis of IVL (1 month later of the initial symptom) for 5 cycles. No significant improvement in symptoms and the patient died from a fulminant cerebral relapse
Jitpratoom et al. (2011) [25]	42 M	Progressive ataxia, motor aphasia, frontal lobe release sign over 2 months. Within 2 weeks, left hemiparesis, altered mental status, and fever of unknown origin	Serum: elevated ESR 89 mm/hr, CRP 64 mg/L, and LDH 873 U/L	CT head: Multiple hypodensities in the bilateral cerebrum and in the cerebellum; MRI head: diffuse bilateral multistage infarction and hemorrhages; MRA head: negative; bone marrow biopsy: negative; random skin biopsy from thigh: IVL	Initially treated with antiplatelet for stroke. For suspicion of vasculitis, treated with pulse steroids k with mild improvement of symptoms. Died on the 10^th^ day from septic shock before initiating chemotherapy
Muto et al. (2011) [26]	74 F	Right-sided paresis over 12 weeks, loss of appetite, weight loss of 8 kg	Serum: MPO-ANCA positive 414 U/ml, ESR 116 mg/dl, CRP 1.1 mg/dl, thrombocytopenia; after 4 weeks, CRP elevated to 16 mg/dl, anemia, thrombocytopenia, elevated LDH 503 IU/l, elevated sIL-2R 4818 U/ml and normalized MPO-ANCA; peripheral blood smear: hemophagocytosis	MRI brain: subacute bilateral scattered infarcts; EMG: multiple mononeuropathies; repeat MRI brain: new stroke in the corpus callosum, bilateral cerebellar and cerebral hemisphere; CT abdomen: hypodensity in bilateral kidney and spleen; FDG-PET/CT: increased uptake in the spleen, liver, sternum, right thyroid, para-aortic lymph node, bone marrow; random skin biopsy from lower abdomen and thigh: IVL; bone marrow biopsy: CD20+ lymphoid cells	Suspected microscopic polyangiitis and treated with pulse steroids for 3 days followed by 50 mg per day along with cyclophosphamide for 3 and half months. After a diagnosis of IVL on the 14^th^ day of re-admission started on CHOP-R every 21 days. After the 5^th^ cycle, the patient died of aspiration pneumonia
Sengupta et al. (2011) [27]	64 F	Presents with gait unsteadiness. 6 months later, left leg weakness and gait unsteadiness. 2 months later, had dysarthria, progressive gait imbalance, and intermittent nausea/vomiting. A few weeks later came with a fall	NA	MRI brain: left inferior cerebellar infarct; MRI brain after 6 months: new infarcts in bilateral cerebellum; multiple MRI brain thereafter: new numerous new punctuate infarcts of both acute and subacute onset in the cerebellar hemispheres, pons, internal capsule, splenium of the corpus callosum, and centrum semiovale bilaterally; conventional angiogram: negative; CSF: normal; bone marrow biopsy: normal; brain biopsy of right occipital cortex with stroke: IVL	Initially treated with antiplatelets. After the second stroke antiplatelet was switched. After the third stroke, dual antiplatelet therapy was used. Later verapamil and cilostazol were added for possible vaso-spasm which caused orthostatic hypotension. After diagnosis of IVL- High dose methotrexate and CHOP-R After 3 cycles, attention span and balance improved
Marino et al. (2012) [28]	71 M	Transient repetitive acute consciousness disturbances, loss of weight, fever, nocturnal itching, dizziness, and gait disturbances over 4 months. Presented with new transient motor aphasia followed by consciousness disturbances, right-sided paresis, ocular bobbing, and seizure. Then developed a rapid progressive impairment of consciousness until the coma	CSF analysis: increased total proteins, cytological analysis revealed the presence of several lymphoblastic cells	Initial MRI brain: unremarkable; EEG: left temporal theta waves during hyperpnea; repeat MRI brain: acute ischemic lesion in the left pons; repeat MRI brain after new symptoms: diffuse tumefactive hemorrhagic leukoencephalopathy with patchy restricted diffusion and enhancement; CT abdomen: lumbo-aortic lymph node of 2 cm in diameter with benign features; brain autopsy: multiple localizations of intravascular large B-cell lymphoma (CD20+, CD3−, CD5−, MUM1+, CD10−) in the brain, adrenal glands, kidneys, stomach and lungs, brain microhemorrhages	The patient developed a rapid progressive impairment of consciousness until coma. Died 8 days after admission
Momota et al. (2012) [29]	67 F	Presented with left-sided weakness. Post steroid therapy developed consciousness disturbance and left-sided hemiparesis	Serum: elevated LDH at 256U/L, elevated sIL-2R at 5896U/mL; CSF: cytology negative for malignancy	MRI brain: multiple ischemic lesions; repeat MRI brain: progressive new lesions; MRA: negative; FDG-PET: negative; bone marrow: negative; needle brain biopsy: within vessels accumulation of tumor cells positive staining for CD31 & 20, MUM-1. Negative for CD5,10 and BCL-6	Treated with steroid administration (betamethasone 4–8 mg/body) with minimal improvement then with 3 cycles of high-dose methotrexate chemotherapy followed by whole-brain radiation therapy. Despite improvement noted on MRI, the patient’s general condition declined. Died 6 months post-disease onset
Cruto et al. (2013) [30]	57 F	Acute onset left leg numbness and weakness. Three months later left arm weakness and left visual field blurry vision	Serum: elevated LDH 290 U/L, normal ESR/CRP; CSF: negative	MRI brain: subcortical multifocal T2 and DWI hyperintense lesions, ADC hypo intensity and contrast enhancement in some lesions suggesting watershed infraction; MRA: normal; TTE: PFO without ASA; brain biopsy: proliferation of large atypical lymphoid cells in the vessels producing luminal obstruction surrounded by necrotic lesions consistent with cerebral infarction; Immunohistochemistry: + CD20	3 days of 1 gm IV steroids Vit K antagonist. After diagnosis of IVL, R-CHOP plus MTX followed by cranial radiotherapy. Eight months after the diagnosis neurological state remains stable, without recovery (MRS 3)
Haninger et al. (2013) [31]	75 F	Multiple episodes of slurred speech, spastic movement of upper extremities, and facial droop lasting for 45 minutes. Developed new seizures during the hospital stay. Readmitted after 2 weeks with confusion and delirium	NA	MRI brain: hemorrhagic infarct in left frontal lobe with focal bilateral parietal lobe infarct; EEG: potential epileptogenic activity in left frontal region; MRI/CT head after 2 weeks: new infarcts, mass effect in right ventricles and MLS to left; brain biopsy: diffuse proliferation of malignant lymphocytes within the lumina of both large and small vessels with luminal obstruction; immunophenotyping: +CD20, -CD5, +BCL-2, +MUM-1, -CD10, -BCL-6	The patient died after 4 weeks of initial symptoms
Hung et al. (2014) [32]	70 F	Mild aphasia, right visual field defect, acalculia, and memory impairment. 8 weeks later developed acute onset dressing apraxia and left-sided neglect. Five weeks later, acute global aphasia, drowsiness, right hemiparesis, and left gaze deviation	Serum: elevated ESR 88 mm/hr, ANA 1:160; CSF: elevated protein 152 mg/dl and IgG level 25 mg/dl; CSF immunofixation electrophoresis: monoclonal gammopathy of IgG with lambda light chains	MRI brain: Left parietal-occipital DWI hyperintense and ADC hypointense lesion; MRI brain after 8 weeks: multiple bilateral ischemic infarcts with mild hemorrhagic conversion; MRA: mild stenosis of bilateral ICA, MCA, ACA, and left VA; MRI brain after 13 weeks: new multistage bilateral infarcts with unchanged MRA; conventional angiogram: multiple focal segmental narrowing suggesting vasculitis; brain biopsy: discohesive cells with high nucleus to cytoplasm ration in the vascular spaces which were immunoreactive to CD45 and CD20 antibodies suggesting IVL	Initially started on aspirin. Died from septic shock one month after the pathological diagnosis
	65 F	Acute decreased consciousness (stuporous), right gaze deviation, left central facial palsy, and left hemiparesis. 3 weeks later acute onset slurred speech, right-sided weakness	NA	MRI brain 24 hours after initial symptoms: right frontal acute infarct; MRA head: negative; repeat MRI brain: new bilateral hemisphere infarction; brain biopsy: enlarged discohesive atypical cells with high nucleus-cytoplasm ratio, logged in vascular spaces and positive for CD20, MUM-1, BCL-6 confirming IVL	Received IV TPA 85 minutes after onset of the symptom. After pathological diagnosis after 1 month of symptom onset, whole brain radiation, R-CHOP X 6 cycles for 8 months which stopped disease progression with mild clinical improvement. Went stuporous on the 10^th^ month
Prayson et al. (2016) [33]	60 M	Acute onset left-hand weakness and gait disturbance over a week	NA	MRI brain: 10 small infarcts in bilateral cerebrum and cerebellum; DSA: suggestive of vasculitis (no further explanation); brain biopsy: proliferation of large, atypical cells in vessel lumen stained positively with CD20 confirming IVL	Started on warfarin but kept developing infarcts. Treated with steroids and cyclosporin for presumed vasculitis. After pathological diagnosis, treated with anthracycline-based chemotherapy
Usuda et al. (2016) [34]	79 F	Presented with cognitive decline and right-sided weakness. On the 44^th^ day in the hospital, developed a sudden onset of unconsciousness and incomplete paralysis on the right side. Later developed a sudden onset of consciousness disorder and incomplete paralysis on the left side of her body on the 79^th^ day of her hospital stay	Serum: anemia, hypoalbuminemia, elevated CRP, alkaline phosphatase, LDH, γ- glutamyltransferase, phosphorus, blood sugar, and hemoglobin A1c, sIL-2R (3300 U/mL), elevated biliary enzymes	MRI brain: multiple ischemic lesions including in the left temporal-parietal region and bilateral cerebellum which were T2 hyperintense; serial MRI brain: T2WI showed a new ischemic lesion in the left anterior region and later in the right frontal region; MRA head: mild stenosis of the bilateral internal carotid arteries, middle cerebral arteries, and anterior cerebral arteries; CT CAP: multiple bilateral nodular lesions in the lung and multiple tumor lesions in the liver; autopsy: atypical lymphoid cells within vessels of brain, lung, and liver	Aspirin 200 mg and ticlopidine 100 mg for suspected ischemic lacunar stroke. Anticoagulant therapy by argatroban, heparin, and warfarin for suspected recurrent ischemic stroke. Treated with antibiotics for cholecystitis. Died on the 123^rd^ day in the setting of multi-organ failure followed by death
Ohya et al. (2017) [35]	68 M	Transient ideomotor apraxia. 3 months post-discharge consciousness worsened after a fall along with left-half spatial neglect and left hemiplegia	Serum: thrombocytopenia, slight anemia, and impaired renal function, elevated LDH at 373 IU/L, CRP of 0.50 mg/dL, elevated sIL-2R: 1322 U/mL; serum on readmission: LDH 2,064 IU/L, CRP 5.7 mg/dl and sIL-2R 1,462 U/mL levels were elevated; CSF: elevated leucocytes (15/uL) and protein 352 mg/dL; cytogenetics: unremarkable for lymphoid cells	MRI brain: small lesions in the bilateral subcortical region of the frontal lobe, which were hyperintense on T2 FLAIR; serial brain MRI (2^nd^ admission): showed more and larger hyperintense lesions in the bilateral subcortical region on DWI and T2; MRA: unremarkable for stenosis in intracranial and carotid arteries; transesophageal echocardiography: 7-mm complicated lesion in the aortic arch; skin biopsy: negative for lymphoid cells; autopsy/brain biopsy: lymphoid tumor cells confined to the small vessels of brain, sleep, bone marrow, kidney and liver	Aspirin was initiated for suspected ischemic stroke. Died on 11-day post 2^nd^ admission
Sharma et al. (2017) [36]	62 F	Episodes of speech difficulty, disorientation, hallucinations, and confusion. Over the next 2 months, cognitive status and language dysfunction deteriorated and developed recurrent seizures, generalized weakness, and a tendency to fall	Serum biochemical and microbiological tests: unremarkable; CSF: white blood cell counts 7–10/mm3, protein 62–85 mg/dL, and glucose 49–51 mg/dL, all normal or borderline elevated values; CSF cytology and flow cytometry studies: normal	Initial MRI brain: two enhancing lesions in the left frontal operculum and right parietal cortex. A small hyperintense DWI lesion in the right cerebellum indicating possible ischemia; repeat MRI brain 1 month later: near complete resolution of the above lesions; MRI brain 2 yrs later: multiple areas of acute ischemia in the right lateral splenium, left corona radiata, right parietal convexity and left superior vermis, as well as multiple areas of vasogenic edema with contrast enhancement in bilateral hemispheres; conventional cerebral angiography: diffuse but mild distal small vessel irregularities suggestive of atherosclerotic changes in the anterior and posterior circulation; CT chest, abdomen, pelvis and whole body PET: unremarkable for cancer; bone marrow aspirate/biopsy/flow cytometry: unremarkable; brain biopsy: abnormal T-cells (+ CD3,8) consistent with IVL	Treated with intravenous methylprednisolone, dexamethasone, intrathecal methotrexate, and cytarabine. Post-treatment, no improvement was noted on imaging followed by the patient’s demise 1 week later
Yunce et al. (2018) [37]	63 F	Transient repetitive right hand and arm numbness, transient left facial droop, and vertigo over 1 month which progressed in frequency. Discharged twice with similar complaints without significant diagnosis apart from hypothyroidism. Two months later developed episodes of confusion and bilateral lower extremity weakness without bowel or bladder incontinence. Three months later, developed additional symptoms including confusion, disorientation, confabulation, cognitive decline, and lower extremity weakness	Serum: anemia, thrombocytopenia (platelet 80,000 per microliter), elevated LDH of 527 U/L. Thyroglobulin level was >1000 IU/mL; CSF: normal	Initial MRI brain: old left cerebellar lacunar infarction; repeat MRI brain: bilateral multifocal white matter change; MRI spine: negative; conventional angiogram: negative; EEG: bilateral temporal epileptiform discharges; PET/CT was notable for mild diffuse FDG uptake throughout the visualized axial and appendicular skeleton; bone marrow biopsy: normal; T-cell gene rearrangement flow cytometry was normal; random skin biopsy: confirmed the presence of IVL	For suspected stroke patient was started on aspirin and a statin. Levothyroxine for hypothyroidism. Alprazolam for panic attacks. After IVL diagnosis, treated with IVsteroids; R-CHOP therapy was started which led to marked improvement of her symptoms
Lyden et al. (2019) [38]	64 M	Progressive weakness of the lower extremities, constipation, and urinary dysfunction over a period of 4 weeks. Additionally developed confusion, prosopagnosia, dysnomia, color blindness, and lower extremity paraparesis	Serum: elevated LDH, positive beta-2 glycoprotein, a polyclonal gammopathy with elevated gamma component, suspicious for multiple myeloma. Elevated methylenetetrahydrofolate reductase (MTHFR) C677T gene, weakly positive hexagonal phase lupus anticoagulant, reduced antithrombin and protein C activity, elevated factor VIII activity; CSF: normal	Initial MRI brain: multifocal acute and subacute infarcts; initial MRI spine: noncontributory; repeat MRI brain and spine: new cerebral infarction and new spinal infarction at T6-7; CTAP: splenomegaly; cerebral angiogram: negative; bone marrow biopsy: elevated plasma cells of normal morphology and normal flow cytometry; brain biopsy: consistent with "double expressor" (MYC and BCL2) nongerminal center IVL	Started on anticoagulation for a possible embolic stroke. The patient deteriorated and passed away prior to starting IVL treatment
Woo et al. (2019) [11]	60 F	Stroke symptoms (not specified). 3-weeks post anti-platelet therapy patient’s dysarthria worsened and then developed a fever	Serum: elevated serum LDH (517 IU/L); CSF PCR: T-cell receptor (TCR)-γ rearrangement analysis, showed monoclonality suggesting IVL	MRI brain: new multifocal infarctions; MRA brain: revealed no stenosis; CT chest and AP: was remarkable for cancer; Brain biopsy: IVL; immunohistochemical analysis: revealed positive expression of CD20, Bcl-2, Bcl-6, Ki-67, and MUM-1	Initially treated with aspirin and clopidogrel. After IVL diagnosis, chemotherapy for IVL was initiated, and the patient passed away 6 months later due to multi-organ failure
	52 F	Acute right-hand weakness and visual disturbance over a 5-month period	Serum: elevated serum LDH level to 393 IU/L; CSF PCR: immunoglobulin heavy chain (IgH) rearrangement analysis demonstrated monoclonality suggesting IVL	MRI brain: focal hyperintense T2 lesions in the left splenium and right occipital lobe; over the next 2 months. Sequential, acute infarct noted in right MCA, R PCA, and left MCA territories; brain MRI 5 months later: revealed leptomeningeal enhancement with enhanced nodules around the fourth ventricle, which was consistent with meningeal involvement of IVL; MRA: no stenosis; CTAP: wedge-shaped infarcts in kidney and spleen; duplex venous legs: DVT in bilateral tibial veins	Initially treated with aspirin and warfarin considering embolic stroke. Later after diagnosis of IVL treated with rituximab-based chemotherapy regimen. On a 5-month follow-up, the neurological status remained unchanged
Chen et al. (2020) [39]	47 F	Right-sided paresthesia, worsening migraine with aura, somnolence, and cognitive decline over 1 month. Symptoms improved transiently followed by worsening headache, vomiting, lethargy, and episodic left facial droop and later progressed to coma	Serum: elevated LDH at 45 0U/L. Low total protein; CSF: marked protein elevation (140 mg/dL) without pleocytosis	MRI brain with contrast: non-specific (T2FLAIR) hyperintensities in the subcortical white matter; serial MRI brain: progressive new development of restricted diffusion lesion on deep white matter, progressive T2 hyperintense lesions, right hemispheric IPH, cerebral edema with mass effect and enhancing lesions; cerebral angiography/MRA: irregular corrugated and beaded appearance of the cervical carotid arteries, thought to be consistent with chronic fibro- muscular dysplasia; repeat cerebral angiography: new right cervical internal carotid artery dissection in the distal extracranial portion as well as progression of luminal irregularities indicative of progressive vasculopathy; MRA: new bilateral intracranial vertebral artery dissection; high-resolution vessel wall MRI: no arterial wall enhancement to suggest vasculitis; FDG-PET scan of the brain: unrevealing; brain biopsy (right temporo-parietal): neoplastic lymphocytes distending small cortical vessels; immunohistochemistry: CD20+ B-cell and labeled widely for MIB-1	Initially treated with a five-day trial of intravenous methylprednisolone followed by a prednisone taper. Then (R-CHOP) therapy along with decompressive craniectomy. Therapy halted the progression of the disease and improved modified Rankin Scale to 1
Chwalisz et al. (2020) [40]	56 M	Left facial droop, left-sided numbness, incoordination, left eye visual distortion, and sensory changes over 4 months	CSF: elevated protein 71 mg/dL (range 5–55), elevated immunoglobulin G 7.8 (upper limit of normal: 4.3), and elevated beta-2-microglobulin 1.98 (range 0.7–1.8)	MRI brain: multifocal areas of DWI hyperintense signal involving the white matter of both frontal lobes, the left parietal lobe, the splenium of the corpus callosum, the left temporal lobe, and the left cerebellar hemisphere; repeat MRI brain: enlargement of many of the foci of abnormal DWI signal, compared to previous one; MRA: unremarkable for carotid or vertebral stenosis; optical coherence tomography scans: thickening of the choroid; fluorescein angiography: bilateral delayed arteriovenous and choroidal filling with distal pruned capillary bed, leakage, and window defects more prominent in the left eye; computed tomography of the abdomen and pelvis with contrast: 3 distinct lobulated retroperitoneal masses that demonstrated soft-tissue attenuation with mass effect on the adjacent right kidney; biopsy of perinephric mass: large neoplastic lymphoid cells; immunohistochemistry: CD20+ PAX5+ B cells that coexpress BCL6, MUM1, and BCL2. Negative for CD10, CD21, and CD30. 60% + CMYC. The proliferation fraction was approximately 90% based on Ki-67; brain biopsy (right frontal lesion): large atypical lymphoid cells, with occasional mitoses plugging capillaries in the cerebral cortex; immunohistochemical stains: CD20 and Pax-5 positive tumor cells, extremely high positivity with Ki-67. CD3 shows scattered perivascular and parenchymal mature T-cells	Treated with aspirin 81 mg daily and atorvastatin 10 mg for multi-focal embolic stroke of unknown etiology. Received intercalated high-dose methotrexate and R-CHOP (rituximab, cyclophosphamide, doxorubicin, and prednisone) chemotherapy. Received thiotepa, busulfan, and cyclophosphamide conditioning followed by autologous hematopoietic stem cell transplantation, which was complicated by mucositis, febrile neutropenia, graft-versus-host disease, clostridium difficile colitis, cryptogenic cirrhosis, and severe malnutrition. Died at 13th months after the initial diagnosis of complications of cirrhosis and portal hypertension
Rota et al. (2020) [41]	62 M	Initially had transient aphasia. 1 week later, developed right hemiparesis and a seizure	NA	MRI brain: T2-hyperintense subcortical lesions in the frontal and temporal regions, with negative DWI, suspected inflammatory-vasculitis appearance; total body CT and cerebral angiography: unremarkable; EEG: mild slowing; postmortem brain biopsy: consistent with IVL	Treated with high-dose IV methylprednisolone without improvement. The patient died one 1-month later
Kimura et al. (2020) [42]	80 M	Consciousness disturbances after sudden convulsions	Serum: elevated serum LDH at 1443 U/L, elevated sIL-2R at 1992 U/mL; cerebral bleeding site flow cytometry: monoclonal B-cell lymphocytes	CT head: cerebral hemorrhagic changes MRI brain: numerous microbleeds and superficial siderosis with edematous changes around the hematomas; repeat MRI brain: expanding hematoma; PET: diffuse accumulation in the bone marrow of the limbs and pelvis; brain biopsy of microbleed at right temporal tip: showed CD20-positive cells in small vessels confirming IVL; bone marrow biopsy: atypical lymphocytes in blood vessels consistent with IVL	R-CHOP X 6 cycles and 4 cycles of intrathecal triple therapy with methotrexate, cytarabine, and dexamethasone. Post-chemotherapy neurological symptoms recovered almost completely within 91 days; the bleeding on susceptibility-weighted imaging remained unchanged
Richie et al. (2013-2020 [43]	6 cases: median age 67 (55-72)/(4F + 2M)	NA	CSF: all patients had CSF analysis; only 1 demonstrated a modest pleocytosis, 3 had unremarkable results of flow cytometry, and all had at least 1 negative result on flow cytometry	MRI brain: abnormal susceptibility was seen in all patients, occurring in both supratentorial and infratentorial gray and white matter, often punctate or gyri form, inconsistently associated with T2 hyperintensity and/or reduced diffusion. All patients demonstrated abnormal white matter T2 or FLAIR hyperintensities. 5 patients demonstrated reduced diffusion, predominantly in small vessel patterns. 4 patients showed abnormal enhancements, often subtle, parenchymal, and involving gray or white matter. Case1: innumerable subcortical microhemorrhages; case 2: few punctate foci of microhemorrhage on SWI not appreciable on subsequent gradient echo sequences T2*weighted imaging; case 6: lobar microhemorrhages; Biopsy (3 Brain biopsy, 2 skin biopsy, 1 autopsy): IVL	
Anbil et al. (2021) [44]	66 F	Initially had difficulty finding words. 3 months later developed bilateral lower extremity weakness. Post-anticoagulation therapy developed new urinary incontinence and bilateral lower extremity paralysis. 2-weeks later developed right upper extremity weakness	Serum: elevated ANA titer at 1:160, elevated LDH at 458, elevated CRP and ESR; CSF: Elevated protein at 170 mg/dL	CT head: unremarkable; MRI brain: bilateral embolic infarcts; serial brain MRIs demonstrated evolving multiage infarctions that were atypical of embolic etiology; cerebral angiogram: unremarkable for large vessel vasculitis; EEG: unremarkable; whole-body PET scan: hypermetabolic 4-mm cervical lymph node suspicious for nodal metastasis; brain biopsy: focal dilated vessels containing abnormal lymphoid cells, with Immunostaining positivity of PAX5 and CD20 consistent with IVL	Initially treated with apixaban with concern for embolic infarcts. IVIG and methylprednisolone were administered daily with 4 sessions of plasmapheresis, resulting in mild improvement in bilateral plantar flexion. Discharged on prednisone. Referred to oncology for initiation of chemotherapy
Wu et al. (2021) [45]	47 F	6-month history of rapidly progressive dementia along with 3 days of left limb weakness and numbness. Clinical condition improved 2 weeks post-discharge. Later developed further memory decline, calculation ability decline, visuoconstructive ability decline, disorientation, left-sided paralysis, dysarthria, seizure, and eventually progressed to coma	Serum: initially normal LDH and routine blood test. On readmission, LDH was elevated (338 U/L); initial CSF: leukocyte count (20106 /L) and protein (0.89 g/L) with normal CSF glucose and chlorine; CSF cytology: no malignant cells, negative infectious and autoimmune panel; repeat CSF: elevated leukocyte count (10106 /L) and protein (1.77 g/L) with normal glucose and chlorine; CSF samples were negative for malignant cells	MRI brain: bilateral multiple hyperintense lesions in periventricular white matter, centrum semiovale, corpus callosum, and cerebellum on T2 FLAIR; MRI spine: within normal limits; repeat MRI brain: enlarged and increased lesions on T2/DWI with open ring enhancement; MRA: unremarkable; bone marrow and random incisional skin biopsy: unremarkable; brain biopsy: occlusion of the small vessels by neoplastic cells with prominent nucleoli, CD2+, consistent with IVL	Treated with IV methylprednisolone (1000 mg/d for 5 days, 500 mg/d for 3 days, 250 mg/d for 2 days, and 125 mg/d for 1 day), with subsequent oral methylprednisolone (20 mg/d) for 1 month and azathioprine (100 mg/d) for maintenance. Died of brain herniation after 10 days of admission
Zhong et al. (2023) [46]	39 F	3 months of headache, fever, anemia, confusion, disorientation, combative behavior, severe headache, and recurrent fever	Serum: thrombocytopenia, renal insufficiency, anemia, thrombocytopenia, elevated LDH >9000 U/L, elevated ANA, anti-dsDNA, SSA, SSB, anti-cardiolipin glycoprotein, and lupus inhibitory; CSF: normal	MRI brain: abnormal T2/FLAIR signal in the superior vermis, enlarged pituitary gland; bone marrow biopsy: hypercellularity without malignancy; brain biopsy: pituitary and cerebellar vasculature contained lymphoma cell associated with clots; autopsy of the body showed extensive disease with involvement of every major organ system and extensive intravascular involvement	Trial of steroids, mental status transiently improved. Died 3.5 months after initial presentation
	61 M	6 months of fever, fatigue, extremity weakness, peripheral edema, intermittent confusion, increased somnolence, tingling, numbness in bilateral lower extremities with bilateral foot drop, low-grade fever, weight loss, fatigue, ascites, and pleural effusion	Serum: anemia, positive ANA, hypoalbuminemia, elevated ESR	MRI brain: mild periventricular white matter ischemic changes of aging. No sign of acute infarction or enhancing lesions. No intracranial hemorrhage; bone marrow biopsy: small clonal population of B cells; brain biopsy: striking infiltration of lymphoma cells within the lumina of intramuscular blood vessels; immunocytochemical identification of CD20, CD79a, CD5 positive atypical large B-cell population in clumps	Responded to R-CHOP therapy X 6 cycles. Relapsed one year after the initial presentation and was treated with MIME and rituximab, which improved his symptoms and functional status
	69 F	10 days of lethargy, intermittent. fever, ataxia, headache, vision changes, progressive confusion, right-sided weakness, and gaze deviation. 3 months history of transient focal weakness, neglect, and expressive aphasia	Serum: anemia, thrombocytopenia hypoalbuminemia, elevated ESR, elevated LDH; CSF: pleocytosis with elevated protein	MRI brain: dynamic change with recurrent multiple foci restricted diffusion in the bilateral frontal and parietal lobes, and thalamus, confluent white matter hyper-intensities involving multiple cortical and sub-cortical locations; brain biopsy and autopsy: revealed CD20-positive atypical B-cell within the lumina of the vessel in brain tissue	Trial of steroids with transient improvement of symptoms Died within 3 months of initial presentation
	60 M	2 months of new-onset recurrent seizure, progressive confusion, severe headache, right arm weakness, and right facial twitching	Serum: elevated ESR and CRP; CSF: no abnormalities including flow cytometry	MRI brain: dynamic changes with recurrent multiple foci restricted diffusion involving multiple watershed distributions. Diffuse patchy T2/FLAIR signals involving both white matter and cortical areas, with some enhancement. Different stages of SAH and IPH; brain biopsy and autopsy: extensive lymphoma cells within the lumina of blood vessels with positive CD20 immunostaining	Trial of steroids with transient improvements of symptoms. Died within 3.5 months of initial clinical presentation
	68 F	6 months of fatigue, confusion, ataxia, seizure, progressive gait disturbance, and ataxia	Serum: elevated LDH; CSF: Mild protein elevation, no pleocytosis	MRI brain: patchy cortical and sub-cortical areas of T2/FLAIR signal abnormalities with corresponding patchy enhancement within the frontal lobe and cerebellum; bone marrow biopsy: negative; brain biopsy: IVL with CD20+ cells within the small vessels and capillaries throughout	Treated with MTX and rituximab X 8 cycles and monthly maintenance MTX X 12 cycles. Survived for 23 months and still alive till publication

Primary Outcomes

**Figure 2:**
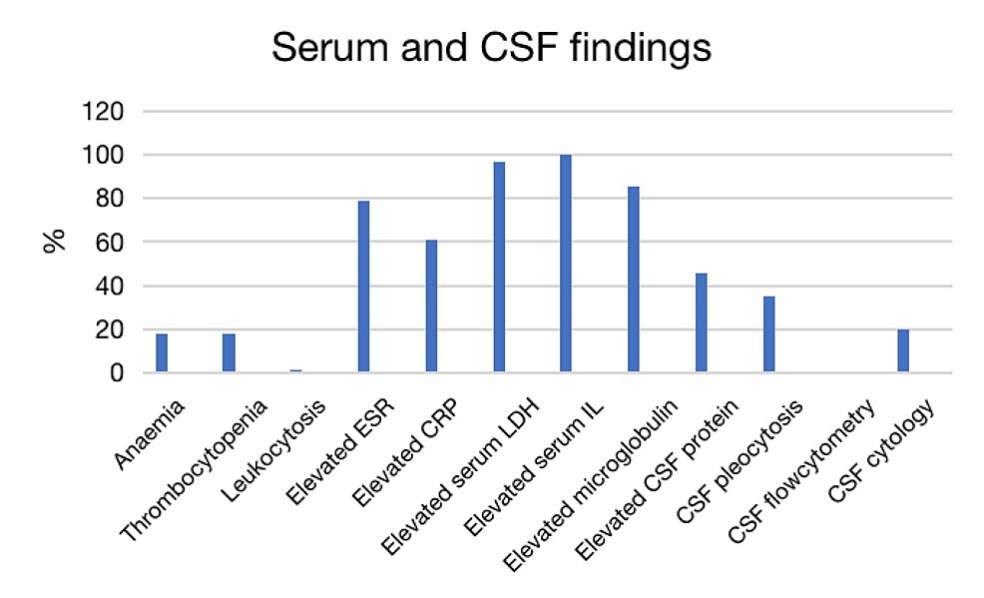
Frequency of serum and CSF analysis findings of all cases CSF: cerebrospinal fluid, ESR: erythrocyte sedimentation rate, CRP: C-reactive protein, LDH: lactate dehydrogenase, IL: interleukin

Classical lateralizing stroke symptoms were noted in only 69.2% of cases (n=52). B-symptoms were present in 19.6% of cases (n=51). Other non-lateralizing symptoms were noted in 92.3% of cases (n=52) which commonly included altered sensorium (30.8%), rapidly progressive cognitive impairment (23.1%), seizures (21.6%), gait disturbances (19.2%), generalized weakness (15.4%), and paraplegia (13.5%) (Figure 2).

Common hematological abnormalities included elevated lactate dehydrogenase (LDH) (96.6%; n=29), elevated erythrocyte sedimentation rate (ESR) (79%; n=19), and elevated C-reactive protein (CRP) (61.1%; n=18). Elevated serum soluble interleukin-2 receptor (sIL-2R) was noted in all six cases (100%) among whom interleukins were tested [16,26,29,34,35,42]. Bergmann et al.'s study showed that one of the serum alfa-1 and 2 globulin or gamma-globulin were elevated among its four cases [13]. Serum beta-2 microglobulin was elevated in one case [16], while CSF beta-2 microglobulin was elevated in another case [40]. Thrombocytopenia was noted in 17.9% of cases (n=56) and anemia in 18.2% of cases (n=55). None of the cases had leukopenia.

**Figure 3:**
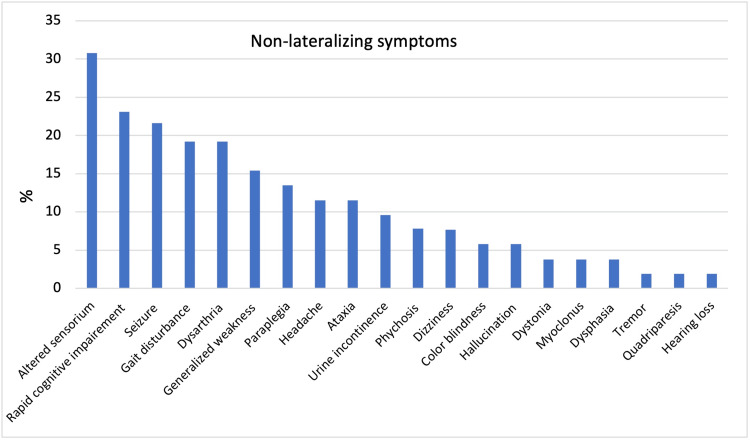
Frequency of non-lateralizing symptoms of all cases

Common CSF analysis findings were elevated protein (45.7%; n=35) and pleocytosis (35.3%; n=34). CSF flow cytometry was done in eight cases, which were all unremarkable. Richie et al.'s study showed at least one negative result on flow cytometry in six cases, and three cases had unremarkable results [43]. Also, CSF flow cytometry of a case from Zhong et al. and Sips et al. showed no evidence of malignancy [20,46]. Kimura et al. reported monoclonal B-cell lymphocytosis in flow cytometric analysis from cerebral bleeding sites [42]. CSF cytology was tested in five cases, among whom only one case cytology showed several malignant lymphoblastic cells [28], while others were negative for malignant cells [16,21,29,36,45]. CSF clonal immunoglobulin heavy chain (IgH) gene rearrangement was noted in two cases that are diagnostic of CNS B-cell lymphoma [11]. Frequencies of significant serum and CSF findings are shown in Figure 3.

All cases had either a head CT and/or MRI demonstrating stroke-like lesions. Fifty-three cases of brain MRI showed either diffusion restriction (39.6%) or abnormal T2-lesions (56.6%), which were mostly located in the subcortical regions (58.5%). The dynamic pattern of MRI diffusion-weighted imaging (DWI)/T2 lesions was noted in multiple studies (n=23) where serial neuroimaging was performed [11,12,15,16,18,20-24,26-29,31,32,34-36,38-40,44,45]. Cerebral hemorrhages were noted in 11.3% of cases (n=53) [19,25,28,39,43,46]. About 30.2% of cases had enhancing lesions in brain MRI [11,13,15,16,18,30,36,39,43,45,46]. Cerebral vessel imaging was mostly unremarkable in 72.7% of cases (n=33), while predominant abnormalities include multifocal stenosis (21.2%) [20,23,32-34,36,39], while two cases had large/medium vessel occlusion [12,19], and one case had distal M1 aneurysm [19]. In electroencephalogram (EEG) findings, background slowing was commonly noted (64%; n=12), while two cases had epileptiform discharges [31,37].

Forty-five cases had follow-up reports, among whom 71.1% died. Only 30.8% of patients were treated with R-CHOP chemotherapy, of whom 25% died. Other therapies included steroids (39.7%) and other chemotherapy (27.6%), along with anti-platelet therapies that proved to be ineffective, leading to death.

Discussion

Our review of 58 cases of IVL with associated stroke first suggests an atypical clinical presentation, unlike classical stroke syndromes. Only 69% of cases had classical lateralizing symptoms to suggest a clinical marker for acute stroke, while the majority had admixed non-specific, non-lateralizing symptoms including altered sensorium, rapidly progressive cognitive impairment, and seizures. There was a high mortality rate among these patients (71%), mainly due to diagnostic challenges and a lack of timely, proper treatment. We summarized frequently occurring laboratory abnormalities and typical radiological characteristics that can aid in the early diagnosis of this fatal disease. During serum work-up, we noted a high frequency of elevated ESR, CRP, sIL-2R, microglobulins, and LDH levels.

Inflammatory markers like ESR and CRP have long served as diagnostic markers of malignancy. CRP had 46.1% sensitivity and 75.4% specificity, and ESR had 43.6% sensitivity and 75.6% specificity to predict various cancers [47]. Ponzoni et al. reported 43% of cases with IVL with elevated ESR [48]. A retrospective study reported that 96% of cases of IVL had elevated CRP levels [9]. In our study, limited data were available for inflammatory markers; however, among reported cases, 79% and 61% cases had elevated ESR and CRP, respectively. Though these are highly non-specific markers for IVL, their persistently elevated levels can serve as an adjunctive to suspect IVL.

sIL-2R is the serum-detectable form of IL-2R, which is found on the lymphocyte membranes, contributing to their activation and proliferation. Its levels elevate in patients with DLBCL, including IVL. With the cutoff of 1104 U/ml, the specificity is 80%, and with a higher cut-off value of 1500-2000 U/ml, the specificity increases to 87-93%, with a positive likelihood ratio of 4.97 [49]. Its sensitivity lies around 35%, even with a higher cut-off value in diagnosing DLBCL since it is detected in most hematolymphoid neoplasms [49]. Since IVL is more associated with stroke-like lesions, sIL-2R levels are of higher yield in the diagnosis. There was a limited sample size for sIL-2R testing in our study; however, in all cases, its level was elevated. It emphasizes that elevated sIL-2R levels can be of high diagnostic utility.

Elevated levels of LDH are a sign of rapid cell turnover, which occurs with lymphoma. The sensitivity and specificity of elevated LDH are 47.4% and 86.5%, respectively, for detecting relapse of DLBCL [50]. Brunet et al. reported that 97% of cases of IVL had elevated LDH levels [9]. Similarly, in our study, almost 97% of cases had elevated LDH levels, which thereby can serve as a key marker for the diagnosis of IVL.

Like LDH, beta-2 microglobulin levels can increase in blood, urine, and CSF in lymphoma due to rapid cell turnover and can serve as a powerful diagnostic and prognostic marker for IVL [51]. Concurrent with the findings, in our study, serum and CSF microglobulin levels were elevated in almost all cases among whom it was tested.

Other hematological abnormalities, like thrombocytopenia and anemia, were noted less frequently in our study, similar to the previous study [48]. However, a study by Brunet et al. suggested a high frequency of anemia and leukopenia among IVL cases [9].

Lymphoma with false-positive perinuclear anti-neutrophil cytoplasmic antibodies (MPO-ANCA) is frequently reported as well. There might be a co-occurrence of vasculitis (biopsy-proven) and lymphoma [26]. Muto et al. reported a case of false-positive MPO-ANCA and IVL, which was thought to be associated with immune dysregulation [26].

Brunet et al. studied 38% of cases of IVL with elevated CSF protein levels [9]. In our study, approximately 46% of cases had elevated CSF protein, which serves as a marker of inflammation. CSF flow cytometry was noted to be unremarkable in our study, while only one case had positive cytology findings among the five tested cases. There is no dedicated study performed yet analyzing the sensitivity and specificity of CSF flow cytometry and cytology in IVL cases, yet Brunet et al. found no cases with cytology suggesting malignant cells among 29 cases of IVL [9]. This suggests a high likelihood of false negative results from CSF flow cytometry or cytology in diagnosing IVL. Therefore, IVL should not be excluded from the differential solely based on these negative findings. However, the sample size of tested cases is smaller in our study, and further studies must be conducted on this topic.

CSF clonal IgH gene rearrangement through PCR analysis has a high specificity of 97% and sensitivity of 54% in the case of CNS lymphomas, with positive and negative predictive values of 93% and 74%, respectively. This test can serve as a significant marker in the diagnosis of CNS lymphoma as well as monitor the therapeutic efficacy given the likelihood of a high false-negative rate of cytology [52]. In our case, only one case was tested and diagnosed with IVL based on clonal IgH gene rearrangement.

Our study further suggests a significant number of cases of EEG diffuse background slowing, which is an indicator of encephalopathy. This is a unique finding, unlike typical stroke cases where focal slowing is usually noted [53].

Among brain MRI markers, the topography of the DWI/T2 lesions was typical in IVL cases, mostly located in the subcortical regions. There is a dynamic pattern in the appearance and resolution of DWI/T2 lesions, with new ones emerging over time. As per Baehring et al., during the acute phase, DWI is highly sensitive, capturing both infarcts and possible tumor cell infiltration. In chronic stages, T2-weighted sequences are more informative, showing subacute infarcts or lymphoid collections, primarily subcortical. Contrast enhancement may appear around DWI/T2 lesions over time due to infarct sequela and blood-brain barrier disruption [15]. Symmetrical confluent white matter lesions that can be seen in IVL can be differentiated from ischemic leukoraiosis or Binswanger’s disease from associated DWI hyperintensity in IVL, which is absent in the case of ischemic leukoraiosis [54]. Due to the involvement of small vessels and non-eloquent areas, initial scans might show T2 changes with or without DWI changes as per the age of the infarcts, where all the lesions might not localize to the presenting symptoms.

Further blood flow dynamics may be unaffected due to the prime involvement of small vessels, and therefore perfusion studies tend to be unremarkable [14]. Other neuroimaging modalities that can be useful in early diagnosis include an 18-fluorodeoxyglucose (FDG-PET) scan [23]. Yamada et al. noted high FDG uptake at the lesion site secondary to IVL [23].

Finally, first-line chemotherapy for IVL includes R-CHOP therapy. In our study, the lowest number of cases received R-CHOP therapy, mostly due to post-mortem diagnosis, concurring with a high mortality rate. A quarter of patients on R-CHOP therapy died, which can be due to delayed diagnosis, high disease burden, or late initiation of treatment. Other chemotherapy and anti-platelet therapies were ineffective, leading to the death. Therefore, early initiation of R-CHOP therapy should be the cornerstone of treatment in patients with IVL-associated strokes.

Limitations

The limitations of our study include a retrospective study design and being able to include only case reports, limiting the sample size and control group. We acknowledge the selection and publication bias associated with this study design. There was limited, complete data on the variables that we intended to study. This further limited our sample size, which might have skewed the values of certain variables. Further large-scale studies need to be conducted to address these issues.

## Conclusions

This comprehensive review suggests that IVL-associated strokes carry a high mortality rate. This is largely due to the challenges of diagnosing the condition promptly and administering timely and proper treatment. Key indicators that can aid in the early diagnosis include a clinical syndrome consisting of B-symptoms and a myriad of non-lateralizing neurological symptoms, a dynamic pattern of DWI/T2 lesions primarily located in the subcortical region, and elevated levels of serum LDH, ESR, CRP, sIL-2R, microglobulins, and CSF protein. CSF PCR analysis with IgH gene rearrangement can be a crucial diagnostic test, apart from the tissue biopsy. Further studies are warranted to develop a predictive scoring system or diagnostic criteria for IVL-associated stroke.

## References

[1] Murase T, Yamaguchi M, Suzuki R (2007). Intravascular large B-cell lymphoma (IVLBCL): a clinicopathologic study of 96 cases with special reference to the immunophenotypic heterogeneity of CD5. Blood.

[2] Harris NL, Jaffe ES, Diebold J (2000). The World Health Organization classification of neoplastic diseases of the haematopoietic and lymphoid tissues: report of the Clinical Advisory Committee meeting, Airlie House, Virginia, November 1997. Histopathology.

[3] PF L, TA J (1959). On the recognition of systematized endotheliomatosis of the cutaneous blood vessels (reticuloendotheliosis?) (Article in German). Hautarzt.

[4] Kinoshita M, Izumoto S, Hashimoto N (2008). Immunohistochemical analysis of adhesion molecules and matrix metalloproteinases in malignant CNS lymphomas: a study comparing primary CNS malignant and CNS intravascular lymphomas. Brain Tumor Pathol.

[5] Ponzoni M, Ferreri AJ (2006). Intravascular lymphoma: a neoplasm of 'homeless' lymphocytes?. Hematol Oncol.

[6] Ferreri AJ, Campo E, Seymour JF (2004). Intravascular lymphoma: clinical presentation, natural history, management and prognostic factors in a series of 38 cases, with special emphasis on the 'cutaneous variant'. Br J Haematol.

[7] Fonkem E, Dayawansa S, Stroberg E (2016). Neurological presentations of intravascular lymphoma (IVL): meta-analysis of 654 patients. BMC Neurol.

[8] DiGiuseppe JA, Nelson WG, Seifter EJ, Boitnott JK, Mann RB (1994). Intravascular lymphomatosis: a clinicopathologic study of 10 cases and assessment of response to chemotherapy. J Clin Oncol.

[9] Brunet V, Marouan S, Routy JP (2017). Retrospective study of intravascular large B-cell lymphoma cases diagnosed in Quebec: a retrospective study of 29 case reports. Medicine (Baltimore).

[10] Kempen JH (2011). Appropriate use and reporting of uncontrolled case series in the medical literature. Am J Ophthalmol.

[11] Woo KA, Yoo D, Jung KH (2019). Intravascular lymphoma as a potential cause of recurrent embolic stroke of undetermined source. J Clin Neurol.

[12] Glass J, Hochberg FH, Miller DC (1993). Intravascular lymphomatosis. A systemic disease with neurologic manifestations. Cancer.

[13] Bergmann M, Terzija-Wessel U, Blasius S (1994). Intravascular lymphomatosis of the CNS: clinicopathologic study and search for expression of oncoproteins and Epstein-Barr virus. Clin Neurol Neurosurg.

[14] Heinrich A, Vogelgesang S, Kirsch M, Khaw AV (2005). Intravascular lymphomatosis presenting as rapidly progressive dementia. Eur Neurol.

[15] Baehring JM, Henchcliffe C, Ledezma CJ, Fulbright R, Hochberg FH (2005). Intravascular lymphoma: magnetic resonance imaging correlates of disease dynamics within the central nervous system. J Neurol Neurosurg Psychiatry.

[16] Imamura K, Awaki E, Aoyama Y, Kondo S, Horie Y, Ohama E, Nakashima K (2006). Intravascular large B-cell lymphoma following a relapsing stroke with temporary fever: a brain biopsy case. Intern Med.

[17] Holmøy T, Nakstad PH, Fredø HL, Kumar T (2007). Intravascular large B-cell lymphoma presenting as cerebellar and cerebral infarction. Arch Neurol.

[18] Iijima M, Fujita A, Uchigata M, Katoo H (2007). Change of brain MRI findings in a patient with intravascular malignant lymphomatosis. Eur J Neurol.

[19] Anda T, Haraguchi W, Miyazato H, Tanaka S, Ishihara T, Aozasa K, Nakamichi I (2008). Ruptured distal middle cerebral artery aneurysm filled with tumor cells in a patient with intravascular large B-cell lymphoma. J Neurosurg.

[20] Sips GJ, Amory CF, Delman BN, Kleinman GM, Lipsey LR, Tuhrim S (2009). Intravascular lymphomatosis of the brain in a patient with myelodysplastic syndrome. Nat Rev Neurol.

[21] Sumer M, Ozon AO, Bakar B, Cila A, Ruacan S (2009). Intravascular lymphoma masquerading as multiembolic stroke developing after coronary artery by-pass surgery. Neurologist.

[22] Boslooper K, Dijkhuizen D, van der Velden AW, Dal M, Meilof JF, Hoogenberg K (2010). Intravascular lymphoma as an unusual cause of multifocal cerebral infarctions discovered on FDG-PET/CT. Neth J Med.

[23] Yamada S, Nishii R, Oka S (2010). FDG-PET a pivotal imaging modality for diagnosis of stroke-onset intravascular lymphoma. Arch Neurol.

[24] Hundsberger T, Cogliatti S, Kleger GR (2011). Intravascular lymphoma mimicking cerebral stroke: report of two cases. Case Rep Neurol.

[25] Jitpratoom P, Yuckpan P, Sitthinamsuwan P, Chotinaiwattarakul W, Chinthammitr Y (2011). Progressive multifocal cerebral infarction from intravascular large B cell lymphoma presenting in a man: a case report. J Med Case Rep.

[26] Muto G, Takahashi Y, Yamashita H, Mimori A (2011). A patient with intravascular lymphoma presenting with cerebral infarction and a high serum MPO-ANCA level. Mod Rheumatol.

[27] Sengupta S, Pedersen NP, Davis JE (2011). Illusion of stroke: intravascular lymphomatosis. Rev Neurol Dis.

[28] Marino D, Sicurelli F, Cerase A, Tripodi S, Cintorino M, Lazzi S, Federico A (2012). Fulminant intravascular lymphomatosis mimicking acute haemorrhagic leukoencephalopathy. J Neurol Sci.

[29] MO H, NA Y, MI Y, SH S (2012). Intravascular lymphoma of the central nervous system presenting as multiple cerebral infarctions. Nagoya J Med Sci.

[30] Cruto C, Taipa R, Monteiro C, Moreira I, Melo-Pires M, Correia M (2013). Multiple cerebral infarcts and intravascular central nervous system lymphoma: a rare but potentially treatable association. J Neurol Sci.

[31] Haninger DM, Davis TA, Parker JR, Slone SP, Parker JC, Jr Jr (2013). Intravascular large B-cell lymphoma presenting as acute hemorrhagic cerebral infarct with delirium. Ann Clin Lab Sci.

[32] Hung LC, Tsai JH, Wu CS, Dai YC, Chen CC, Sung SF (2014). Brain biopsy-proven intravascular lymphomatosis presenting as rapidly recurrent strokes-two case reports. Acta Neurol Taiwan.

[33] Prayson RA (2016). Intravascular lymphoma mimicking vasculitis. J Clin Neurosci.

[34] Usuda D, Arahata M, Temaru R, Iinuma Y, Kanda T, Hayashi S (2016). Autopsy-proven intravascular lymphoma presenting as rapidly recurrent strokes. Case Rep Oncol.

[35] Ohya Y, Osaki M, Sakai S (2017). A case of recurrent ischemic stroke due to intravascular lymphomatosis, undiagnosed by random skin biopsy and brain imaging. Case Rep Neurol.

[36] Sharma TL, Yeaney GA, Soltanzadeh P, Li Y, Cotta CV (2017). Intravascular T-cell lymphoma: a rare, poorly characterized entity with cytotoxic phenotype. Neuropathology.

[37] Yunce M, Muganlinskaya N, Selinger S (2018). Intravascular large B-cell lymphoma presenting as multiple stroke: a case report. Medicine (Baltimore).

[38] Lyden S, Dafer RM (2019). Intravascular lymphomatosis presenting with spinal cord infarction and recurrent ischemic strokes. J Stroke Cerebrovasc Dis.

[39] Chen R, Singh G, McNally JS, Palmer CA, de Havenon A (2020). Intravascular lymphoma with progressive CNS hemorrhage and multiple dissections. Case Rep Neurol Med.

[40] Chwalisz BK, Douglas VP, Douglas KA, Martinez-Lage M, Kelly HR, Cestari DM (2020). Episodic visual distortions and stroke-like symptoms in a 56-year-old man with intravascular lymphoma. J Neuroophthalmol.

[41] Rota E, Pitino A, Pastorino R, Gallesio I, Morelli N (2020). Intravascular large B-cell lymphoma: a forgotten stroke "mimic". Acta Neurol Belg.

[42] Kimura M, Fujiwara S, Tanaka A (2020). Multiple cerebral hemorrhages with microbleeds in intravascular large B-cell lymphoma. J Stroke Cerebrovasc Dis.

[43] Richie MB, Guterman EL, Shah MP, Cha S (2022). Susceptibility-weighted imaging of intravascular lymphoma of the central nervous system. JAMA Neurol.

[44] Anbil S, Fenerty K, Feng Z, Doughty R, El-Farra NS (2021). Intravascular large B cell lymphoma as a cause of multifocal cryptogenic stroke. Am J Med.

[45] Wu M, Lin Y, Huang X, Zhang B (2021). Intravascular large B-cell lymphoma presenting as rapidly progressive dementia and stroke: a case report. Medicine (Baltimore).

[46] Zhong N (2023). Clinical spectrum, diagnosis, and treatment outcome in individuals with intravascular large B-cell lymphoma affecting the nervous system: a case series. Cureus.

[47] Watson J, Salisbury C, Banks J, Whiting P, Hamilton W (2019). Predictive value of inflammatory markers for cancer diagnosis in primary care: a prospective cohort study using electronic health records. Br J Cancer.

[48] Ponzoni M, Campo E, Nakamura S (2018). Intravascular large B-cell lymphoma: a chameleon with multiple faces and many masks. Blood.

[49] Murakami J, Arita K, Wada A (2019). Serum soluble interleukin-2 receptor levels for screening for malignant lymphomas and differential diagnosis from other conditions. Mol Clin Oncol.

[50] Hong J, Yoon HH, Ahn HK (2013). Prognostic role of serum lactate dehydrogenase beyond initial diagnosis: a retrospective analysis of patients with diffuse large B cell lymphoma. Acta Haematol.

[51] Yoo C, Yoon DH, Suh C (2014). Serum beta-2 microglobulin in malignant lymphomas: an old but powerful prognostic factor. Blood Res.

[52] Ekstein D, Ben-Yehuda D, Slyusarevsky E, Lossos A, Linetsky E, Siegal T (2006). CSF analysis of IgH gene rearrangement in CNS lymphoma: relationship to the disease course. J Neurol Sci.

[53] Ag Lamat MS, Abd Rahman MS, Wan Zaidi WA, Yahya WN, Khoo CS, Hod R, Tan HJ (2023). Qualitative electroencephalogram and its predictors in the diagnosis of stroke. Front Neurol.

[54] Kinoshita T, Sugihara S, Matusue E, Nomura T, Ametani M, Ohama E, Ogawa T (2005). Intravascular malignant lymphomatosis: diffusion-weighted magnetic resonance imaging characteristics. Acta Radiol.

